# A Giant Pheochromocytoma Presenting in Pheochromocytoma Crisis: A Case Report

**DOI:** 10.31729/jnma.8027

**Published:** 2023-02-28

**Authors:** Kanchan Bogati, Sunil Baniya, Suresh Thapa, Upendra Krishna Regmi, Niranjan Karki, Manish Pokhrel

**Affiliations:** 1 Patan Academy of Health Sciences, Satdobato, Lalitpur, Nepal; 2 Military Hospital Pokhara, Pokhara, Kaski, Nepal; 3 Department of Surgery, Shree Birendra Hospital, Chhauni, Kathmandu, Nepal; 4 Department of Pathology, Shree Birendra Hospital, Chhauni, Kathmandu, Nepal; 5 Chitwan Medical College, Bharatpur, Chitwan, Nepal

**Keywords:** *case reports*, *headache*, *hypertension*, *pheochromocytoma*

## Abstract

Giant pheochromocytomas are rare tumors, with the majority being clinically silent. Clinically manifesting pheochromocytoma can present with symptoms of catecholamine excess, but nonspecific symptoms and variable clinical patterns of hypertension make it difficult to diagnose. Missing the diagnosis can lead to cardiovascular catastrophes like a pheochromocytoma crisis and even death. We report a 45-year-old woman on antihypertensives, repeatedly visiting a hospital for recurrent headaches finally presented in a hypertensive crisis at an emergency department. Management was started along with an injection of labetalol, which led to an unpredicted abrupt blood pressure fall, and was successfully resuscitated. Imaging and plasma metanephrine studies revealed an underlying giant pheochromocytoma, which was cured after successful surgical resection. A high degree of clinical suspicion, elaborate and focused history-taking, and initial ultrasound imaging can guide us toward the early diagnosis of pheochromocytoma. Before the alpha blockade, beta-blockers should not be used in any cases of pheochromocytoma.

## INTRODUCTION

Pheochromocytoma (PHEO) and paraganglioma (PGL) are neuroendocrine tumors arising from chromaffin cells of the adrenal medulla and extraadrenal autonomic paraganglioma respectively with a combined estimated annual incidence of 0.8 per 100000 person-years.^1^ Giant pheochromocytoma are tumors of size more than 7 cm and are rare. Majority of them are clinically silent.^2,3^ We report a clinically manifesting giant pheochromocytoma previously being missed because of nonspecific clinical features later landed in pheochromocytoma crisis (PCC). PCC is a life threatening complication that requires prompt recognition for correct treatment which is the most challenging.^3,4^

## CASE REPORT

A 45-year-old lady under antihypertensive medication for the last 9 years but discontinued for 7 months due to low blood pressure presented to the emergency department of a tertiary care with complaints of severe headache, nausea, and five episodes of vomiting for 3 days. Headache was associated with sweating, dizziness, tinnitus, fatigue, and palpitations. She had a complaint of headaches since the diagnosis of hypertension but it became more frequent, severe in the last 5 years for which she had repeated hospital visits and admissions and was managed by adjusting antihypertensive medication for rising or fall of blood pressure, using goggles and over the counter analgesic medicines. She was also recently diagnosed as a case of hypothyroidism and peripheral arterial disease and was compliant with medications. No history of chest or abdominal pain. There was no significant family history.

On physical examination, her blood pressure was 200/100 mm of Hg on both arms with a heart rate of 136 bpm and she had pallor. Systemic examination revealed systolic murmur, grade III hypertensive retinopathy of the left eye without any focal neurological deficits. Her blood urea was 50 mg/dl, creatinine was 1.7 mg/dl, and random blood sugar was 194 mg/dl with normal electrolytes. ECG was suggestive of sinus tachycardia. With a diagnosis of hypertensive emergency, we targeted for 10% reduction of blood pressure over the first hour and the next 15% reduction over the next 23 hours. She was given an injection of labetalol 10 mg intravenous over 2 minutes followed by 20 mg after 10 minutes with constant non-invasive blood pressure monitoring. Abruptly, her blood pressure dropped to 80/60 mmHg with a heart rate of 124 bpm. Labetalol was stopped and volume resuscitation started. Her blood urea and creatinine level got raised to 54 mg/ dl and 1.9 mg/dl respectively. She responded well to intravenous fluid resuscitation and achieved normal blood pressure with persisting acute kidney injury (AKI) after infusion of 2 litres of normal saline over 4 hours. She was admitted for further evaluation and management.

**Figure 1:**
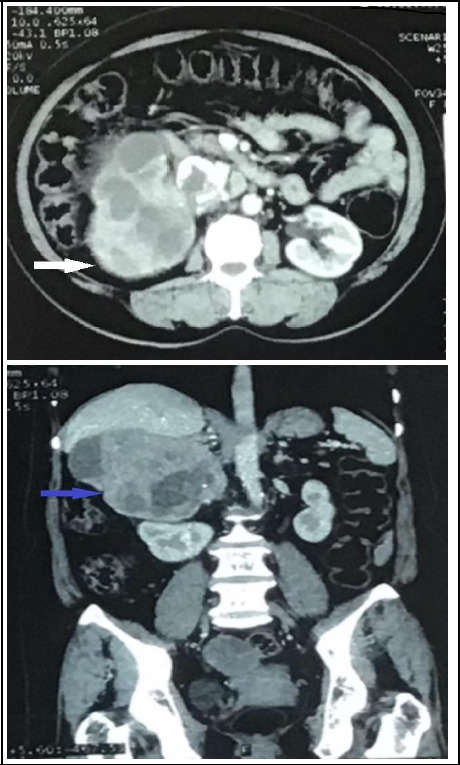
CECT abdomen and pelvis axial section and coronal section show large lobulated heterogeneously enhancing mass lesion (white arrow and blue arrow) in the right retroperitoneal region at the suprarenal location. The right adrenal gland is not separately visualized.

Clinical observation showed that headaches and other associated symptoms were closely associated with the episodic rise of blood pressure. The usual dose of analgesic was unable to relieve her headache. Nitroglycerin infusion was given during sudden rise of blood pressure which was titrated accordingly. It was very difficult to treat her because of the unpredicted rise and fall of blood pressure in the background of acute kidney injury (AKI). With appropriate management, it took 6 days to get a normal range of creatinine. These clinical findings led us towards suspicion of pheochromocytoma. She was under medication for hypothyroidism and was euthyroid. Renal ultrasound for evaluation of hypertension and AKI showed a large mass in the right renal region along with cholelithiasis. After resolution of AKI, the contrast enhanced computed tomography abdomen was done which showed a large lobulated heterogeneously enhancing mass lesion of 14.7x12.9x9.8 cm in the right suprarenal location and right suprarenal gland not visualized separately. It was in contact with the liver with a maintained fat plane but displacing the head of the pancreas anteriorly, displacing inferior vena cava (IVC) with an indistinct fat plane and no abdominal lymphadenopathy (Figure 1). From both clinical and imaging findings, we made differential diagnoses of pheochromocytoma, adrenal medullary hyperplasia, adrenocortical carcinoma, and adrenal metastases. A biochemical assessment was done with plasma-free metanephrine; it showed a value of 250 pg/mL (reference range: less than 65 pg/ mL), which was consistent with pheochromocytoma.

With evidence of right adrenal mass lesion and elevated level of plasma free metanephrine, the diagnosis of pheochromocytoma was made. Magnetic resonance imaging (MRI) was done for better delineation of the tumor mass. Multiple endocrine neoplasia type 2 (MEN 2) was excluded by normal findings of the thyroid gland and neck ultrasound scan, normal serum level of carcinoembryonic antigen (CEA) and calcium, parathyroid hormone for medullary thyroid carcinoma, and parathyroid hyperplasia respectively. Metastasis was ruled out by normal plain CT findings of the neck, chest, and abdomen regions.

**Figure 2:**
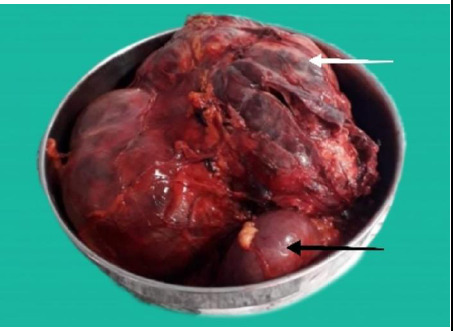
En-bloc resection of the right adrenal gland with tumor (white arrow) with right kidney (black arrow).

Multidisciplinary expert discussion with an endocrinologist, physician, anesthesiologist, and surgeon suggested open right adrenalectomy with possible right nephrectomy and cholecystectomy under general anesthesia considering the tumor's large size, and compression on great vessels and nearby structures, highly vascular and hormonal nature. Preoperative preparation was done for 27 days with initial α-blockade by selective α-1 blocker (prazosin 2.5 mg twice a day for 5 days), then β-blocker was added (metoprolol 25 mg once daily), high sodium diet (>5 gm/day) and intravenous normal saline infusion (100 ml/hr till 5 days before surgery). She underwent successful en-bloc resection of the right adrenal gland and right kidney, tumor resected was 17 x 17 x 10 cm in dimension (Figure 2). The right kidney could not be spared because it was closely associated with the tumor posing the risk of possible pheochromocytoma crisis for excessive handling of the highly vascular tumor. In the same setting, cholecystectomy was also performed. The intraoperative period was uneventful. Postoperatively, she was shifted to intensive care unit (ICU) and kept under observation. Postoperative recovery was also uneventful and shifted to the general ward on the sixth postoperative day.

**Figure 3:**
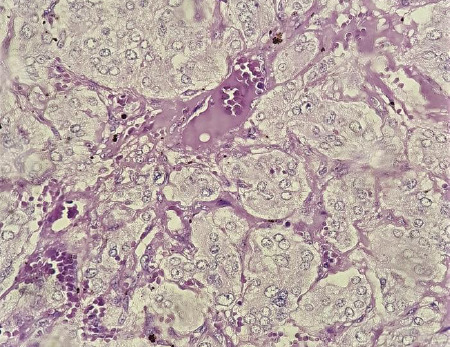
Histology (40X, Hematoxylin and eosin stain) shows tumor cells displaying the characteristic "Zellballen Pattern".

The diagnosis was confirmed after the histopathology report. The gross finding was suprarenal mass measuring 17 x 17 x 10 cm, composed of the solid cystic area with a hard area measuring 7 x 7 x 4 cm, and the capsule was easily stripped off. Microscopy showed tumor cells arranged in characteristic "Zellballen Patterns" separated by a fibrovascular stroma. Tumor cells show mild pleomorphism. They had abundant finely granular amphophilic cytoplasm with round to ovoid vesicular nuclei having prominent nucleoli (Figure 3). Pathological staging was pT2. The immunohistochemical (IHC) profile was consistent with pheochromocytoma, tumor cells stained positively with synaptophysin.

After 67 days of hospital stay, She was discharged on the fourteenth postoperative day with a low dose of an antihypertensive agent (amlodipine 5 mg once daily). At 1 month of follow-up, her report of postoperative plasma-free metanephrine was within the reference range. She was normotensive and the antihypertensive agent was discontinued. She completed 2 years of follow-up with normal reports of plasma-free metanephrine.

## DISCUSSION

Pheochromocytomas are chromaffin cell tumors derived from the neural crest. Many physicians have never encountered this rare disease.^5^ Clinical disease results from symptoms of catecholamine excess in catecholamine secreting PHEO/PGL whereas local compressive symptoms in nonfunctioning PHEO/ PGL.^6^ The symptoms caused by catecholamine excess can mimic more than 30 medical disorders making it a diagnostic challenge.^5^ The lethal consequence of missing the diagnosis is illustrated by the Mayo clinic report of 54 autopsy-proven cases of PHEO, in which 76% were not suspected clinically during life.^7^

The majority of giant pheochromocytomas are usually clinically silent. It is because of failure of production of catecholamines due to possible tumor necrosis, more interstitial tissue than chromaffin cells, and encapsulation of tumor by connective tissue impairing catecholamine release.^2,3^ Here, we have reported a case of giant pheochromocytoma having symptoms of catecholamine excess however, it was missed for a long time because of the nonspecific nature of its clinical features.

Catecholamine excess causes a broad spectrum of nonspecific clinical features. Common manifestations include hypertension, headache, palpitation, diaphoresis, and other less common manifestations are fatigue, nausea, weight loss, constipation, flushing, fever, pallor, tremulousness, chest, and abdominal pain, visual blurring, heat intolerance, hyperglycemia, transitory electrocardiographic changes, polyuria, polydipsia. Hypertension is the most common sign of PHEO/PGL however, it only represents approximately 0.05% of all causes of hypertension.^4,8^ The classic triad of headache, palpitations, and profuse sweating has a reported sensitivity of 91% and specificity of 94% in presence of hypertension.^4^ Hypertension shows a variable clinical pattern, either sustained or paroxysmal or hypertension alternating with hypotension.^9^ During inpatient evaluation, our patient's symptoms were found consistent with paroxysmal hypertension. Literature reports that it is observed in 45% of cases of pheochromocytoma.^9^

PCC is an endocrine emergency associated with significant mortality.^10^ It presents with severe hypertension to circulatory failure and shock with subsequent involvement of one or more organ systems including cardiovascular, pulmonary, neurological, gastrointestinal, renal, hepatic, and metabolic systems. It can occur spontaneously or precipitated by tumor manipulation, trauma, certain drugs (corticosteroids, betα-blockers, metoclopramide, and anesthetic agents), or stress from nonadrenal surgery.^11^ PCC most commonly presents as hypertensive crisis or catecholamine cardiomyopathy.^10^ The incidence rate of PCC given by a retrospective study of patients who underwent surgery for pheochromocytoma was 18%, out of which 36% had prior pheochromocytoma symptoms suggesting PCC can occur with a history of preceding symptoms with or without diagnosis or can be the first manifestation of the tumor.^11^ Literature reports that PCC is associated with a significant mortality rate of 15% emphasizing prompt recognition and administration of appropriate treatment.^10^ In our case, the patient presented with PCC as severe hypertension (blood pressure of 200/100 mmHg in both arms) with AKI with finding of preceding symptoms. We could not recognize this as PCC and treated it for a hypertensive emergency by giving labetalol which led to abrupt blood pressure fall and hypotension however, the patient responded well to intravenous fluid resuscitation. There is a fact that severe hypertension in PCC can also rapidly alternate with hypotension,^10^ we are not sure whether abrupt blood pressure fall was only due to the drug or PCC itself alternating with hypotension with the added effect of the drug in our case. An earlier study had also reported a similar finding: there was cardiovascular collapse after they used labetalol for a hypertensive crisis in an undiagnosed pheochromocytoma during a cesarean section.^12^ Literature strongly recommends not to use beta-blockers before alpha-blockade due to the risk of unopposed alpha-adrenergic action in a patient with pheochromocytoma. Labetalol, a nonselective beta-blocker should also be avoided because though it has some alpha-blocking properties which are insufficiently disproportionate to its beta-blocking effects, therefore can exacerbate the crisis. The use of alpha-blockers (for example phentolamine) is the most widely accepted intervention for PCC.^10^ In our experience, recognition of PCC in a patient presented with a hypertensive crisis is the most challenging.

The diagnosis of PHEO/PGL can only be made by proof of excessive release of catecholamines and anatomical documentation of the tumor.^5^ Superiority of plasma free metanephrines measurement is recently established over urinary metanephrines measurement as a biochemical screening test. Plasma concentration of normetanephrine, metanephrine, or methoxytyramine more than two times the upper limit of the reference range suggests a high probability of disease. Anatomical imaging by CT or MRI has 100% sensitivity for localizing adrenal PHEO.^13^ 90% of clinically manifested PHEO can be detected by ultrasound,^5^ the importance of which was also emphasized in a case report.^14^ Our case also shows the importance of ultrasound screening in the detection of pheochromocytoma. Metastasis/multiplicity of the tumor should be ruled out prior to surgery by whole-body CT or MRI or functional imaging in all patients with PHEO. Genetic testing is recommended for germline mutation in all patients with PHEO and testing for somatic mutation where tumor material is available.^13^

Surgery is always the therapy of choice in PHEO/ PGL.^5^ Symptoms and hypertension caused by PHEO are curable with surgical resection of the tumor. At least 10-14 days of preoperative medical treatment by α-blocker and/ or β-blocker is required to control hypertension and prevent perioperative cardiovascular complications. With the advantages of minimally invasive techniques, laparoscopic adrenalectomy has replaced the open technique.^4^ However for a pheochromocytoma with a diameter of 5 cm or more, it is recommended to choose a surgical method on an individual basis with tumor characteristics and surgical expertise.^5^ In our case, we had open laparotomy and en-bloc removal of the entire right adrenal gland with tumor along with right kidney because of large tumor size (17x17x10 cm^3^) compressing nearby large blood vessels and being closely associated with the right kidney that could lead to possible disastrous consequences if only the tumor mass removal was attempted. Even after complete resection of PHEO, patients are at increased risk of recurrences. They should have at least 10 years of follow-up but if the tumor size is 5 cm or more, lifelong follow up is recommended with a yearly clinical investigation and laboratory assessment of plasma or urinary metanephrines and methoxytyramine.^13^

Pheochromocytomas are rare tumors and are difficult to diagnose. Patients with hypertension with recurrent headaches should be properly evaluated not to miss the diagnosis and to avoid disastrous consequences like pheochromocytoma crisis. Clinically manifested PHEO can even be detected by ultrasound screening in 90% of cases. Laboratory measurement of metanephrine and anatomical documentation by CT or MRI gives a complete diagnosis. Beta-blockers should not be used before alpha blockade in any case. Successful surgical resection is challenging but only the way that cures the disease, however, recurrences are common. Lifelong follow-up is needed.
